# American Medical Association

**Published:** 1849-06

**Authors:** 


### EDITORIAL DEPARTMENT.

*American Medical Association.*—The *third** annual meeting of the American Medical Association was held at Boston, Mass., commencing on Tuesday, the first day of May, and ending on the evening of the following Friday. The place of meeting, for the first two days, was at the lecture hall of the Lowell Institute. The Legislature having adjourned on the second day, the Association occupied, for the two last days of its session, the Representatives' Hall at the State Capitol.

The number of delegates and members present was about four hundred and fifty, being one hundred and eighty four more than were present at

* We perceive that this meeting has been designated the *second* annual meeting of the Association. The Convention of 1847, at Philadelphia, resolved itself into the Association, after the latter was organized; hence, as it seems to us, that was the *first* meeting. The meeting at Boston, therefore, was the *third* annual meeting.

the meeting at Baltimore, in 1848. Twenty-four of the states of the Union were represented.

Upon the organization of the meeting, Dr. John C. Warren, on behalf of the Massachusetts Medical Society, made a short address, expressing salutations of welcome, and congratulations. Dr. Stevens, President of the Association, followed, by an address of considerable length. The following officers for the ensuing year were then elected:—

*President.*—John C. Warren, M. D., of Mass.

*Vice Presidents.*—Drs. J. P. Harrison, of Ohio; H. H. Maguire, of Virginia; A. Flint, of N. Y.; and R. S. Stewart, of Maryland.

*Treasurer.*—Isaac Hays, M. D., of Philadelphia.

*Secretaries.*—Drs. A. Stille of Philadelphia, and H. J. Bowditch, of Boston.

The reception of the reports of standing committees appointed at the previous meeting, then commenced, the reading of which, and discussions therewith connected, occupied the greater part of three of the four days of the session. These reports are able and elaborate, and, when published, will make a volume, larger, by one half, than the volume of transactions for the preceeding year. The great length of most of the papers, precluded the reading of them entire. Much embarrassment grew out of this circumstance, and not a little time was consumed by discussions whether the reports should be read, and how long the reading should continue.. With a view to obviate the recurrence of a similar difficulty at the next meeting, the Association finally resolved, that, hereafter, an abstract of the contents of each report on scientific subjects should be submitted to the meeting, the reading of which should occupy about an hour. We refrain from any critical remarks upon the several reports, until they shall have been perused in print. They will do honor to the respective committees, to the Association, and to the country.

The able report of the committee on Medical Education (Dr. F. Campbell Stewart, of New York, chairman) alone elicited discussion, as respects the subjects presented. A series of resolutions recommending considerable changes in the system of medical instruction, was submitted, in connection with this report, which, after considerable discussion, in committee of the whole, were, for the most part, laid upon the table. The whole subject was referred to a special committee to report on the following day, the last day of the session. As many of our readers may be desirous to know what final action was taken on matters relating to education, we subjoin the resolutions reported by the special committee just referred to,

(of which Dr. A. H. Stevens was chairman,) and adopted by the Association:—

The committee to whom were referred the resolutions offered by the standing committee on medical education, the letter of Dr. John Watson, of the New York Academy of Medicine, &c., have the honor to report, that they have examined the several documents mentioned, and have agreed to recommend to the Association the adoption of the following resolutions:—

1. *Resolved*, That the Association reiterate their approval of the resolutions, in reference to medical education, adopted by the Convention which met in Philadelphia, in May, 1847, and contained in pages 73 and 74 of the published proceedings of that Convention.

2. *Resolved*, That the attention of medical colleges be again directed to the resolutions of the Committee on Preliminary Education, adopted by the Medical Convention of 1847, and that they be advised to require from the students, in all instances, certificates of due preliminary acquirement prior to graduation.

3. *Resolved*, That physicians generally, throughout the Union, be advised and requested to require of those wishing to become their pupils, evidence of a proper general education, before admission into their offices.

4. *Resolved*, That this Association does not sanction or recognize "college clinics," as substitutes for hospital clinical instruction, and that the medical colleges be again advised to insist, in all instances when it is practicable, on the regular attendance of their pupils, during a period of six months, upon the treatment of patients in a well-conducted hospital, or other suitable institution devoted to the reception and cure of the sick.

5. *Resolved*, That in accordance with a resolution of the American Medical Association, adopted May 4th, 1847, it is earnestly recommended to the physicians of those States in which State Medical Societies do not exist, that they take measures to organize them before the next annual meeting of this Association.

6. *Resolved*, That the State Societies be recommended, after they shall have been organized, to recognize as regular practitioners none who have not obtained a degree in medicine, or a license from some regular medical body, obtained after due examination.

7. *Resolved*, That this Association recommend to the various schools of medicine to meet at Cincinnati before the next meeting of this Association, and present a plan for elevating the standard of practical education.

The discussions pertaining to the above resolutions engrossed most of the last day of the session. Some other topics, of interest, however, were considered, and acted upon, the more prominent of which we will mention. Prof. Ware submitted a paper, on behalf of the Faculty of the Medical Department of Harvard University, setting forth their reasons for not extending the period of instruction by lectures; and advocating the propriety of continuing to adhere to the four months' term. After considerable discussion, it was resolved that this paper be published in the volume of Transactions. In consequence of this resolution, Prof. Wood, of Philadelphia, offered the following resolution, which was adopted:—

Whereas, a document prepared by the Medical Faculty of Harvard University, and appended to the report of the committee on medical education, contains an elaborate defence of the limitation of the courses of medical instruction, in the schools, to four months; and whereas this document has been referred, along with the report of the committee on medical education, to the publishing committee, and, if it be not mistaken by the public as a representation of the views of this Association, may, at least, have the effect of contradicting those views, unless they are properly supported, therefore

*Resolved,* That a Committee be appointed to prepare, at leisure, a statement of the facts and arguments which may be adduced in favor of the prolongation of the courses to six months, and that the statement thus prepared be printed in the forthcoming volume of the transactions of the Association.

Drs. S. Jackson, (Prof.,) J. L. Atlee, and Stille, all of Philadelphia, were appointed the committee provided for in the foregoing resolution.

Prof. Evans, of Illinois, submitted the following resolution, which was passed:—

Whereas, merit should be the test by which one individual is preferred to another, and whereas, the places of profit and honor in our profession should be open to the competition of all, in order that the best selections may be made therefrom,

*Resolved,* That trustees, and others exercising the office of appointing Professors in Medical Schools, be requested to adopt the system of *concours,* or public trials, among the means resorted to for calling out the talent of the profession, and ascertaining the qualifications of applicants.

Prof. E. also announced the occurrence of two resignations in the Faculty of the Rush Medical College, and invited applications from those desirous of being appointed to fill the vacancies.

The proposition to amend the constitution so as to permit permanent members, present, but not as delegates, to vote, as well as participate in the other proceedings, created some discussion. It was at length negatived, and, as it appears to us, with great propriety, since, were the proposed amendment to go into effect, after the lapse of several years, if a meeting were to take place in Boston, New York, or any large city in which a previous meeting had been held, the permanent members residing in that city would be sufficiently numerous to constitute a majority, and control the action of the Association.

After the votes of thanks usual on such occasions, but preeminently appropriate and called for on this occasion, the Association adjourned on Friday evening, *sine die,* having previously designated *Cincinnati* as the place of meeting, on the first Tuesday of May, 1850.

The following are the names of the chairmen of standing committees for the present year:—

*Medical Sciences,* Dr. Ware, of Boston; *Practical Medicine,* Dr. J. K. Mitchell, of Philadelphia; *Surgery,* Dr. R. D. Mussey, of Cincinnati; *Obstetrics,* Dr. Prioleau, of Charleston, S. C.; *Medical Education,* Dr. Roby, of Md.; *Medical Literature,* Dr. A. Stille, of Philadelphia; *Publication,* Dr. J. Hays, of Philadelphia; *Indigenous Medical Botany,* Dr. E. Ives, of New Haven; *Forensic Medicine,* Dr. A. H. Stevens, of New York; *Hygiene,* Dr. J. M. Smith, of New York.

It remains to notice some of the social events connected with this meeting of the Association. And here, disclaiming all pretensions to rhetorical powers adequate to such a theme, we must relinquish any attempt to do justice to our Boston brethren, and to our own feelings. On the first evening of the session, the members, with a few other guests, were entertained by Drs. John C. Warren, and Jacob Bigelow, at their respective residences. On Wednesday evening a splendid collation was prepared at the Revere House, which the members of the Association attended by invitation of the physicians of Massachusetts. The guests were welcomed by Dr. Jacob Bigelow, on behalf of the physicians of Mass., and, also, by Hon. John P. Bigelow, Mayor of the city of Boston. The walls of the entertainment Hall were covered with portraits of eminent medical men deceased. The tables were loaded with viands and delicacies, and decorated by a profusion of rich bouquets. The artificial exhilaration of wine would have been quite superfluous, and its exclusion was neither regretted, nor felt. Although it was announced by the committee of arrangements that there would be no speaking, the exuberance of genius and mirth could not be entirely restrained, and the festivities of the evening did not close without some sparkling effusions. We could wish that, for once, our pen, accustomed, as it is, only to the tame duty of medical journalism, might be invested with a graphic power adequate to a faithful delineation of this memorable social occasion; yet, we are consoled in the consciousness of incompetency for so agreeable a task, by the reflection that the regrets of our absent brethren might be enhanced by a vivid description. Fortuneately, the faculty of enjoyment and appreciation of the actual, is not compromised by inability to reproduce, for others, sources of pleasure, by an ideal representation.

On Thursday evening an invitation was extended to the Association to visit, at his splendid mansion, Hon. Abbott Lawrence, a gentleman widely known for his munificent liberality for scientific objects; and on the same evening the same hospitalities were proffered by Drs. Hayward and Homans, at their respective residences.

In addition to the foregoing social attentions, the freedom of the city, as regards all its public institutions, and also those of the vicinity, inclusive even of places of amusement, were tendered to each member, the card of membership, serving as a ticket for gratuitous admission to them.

In fine, the courtesies, and hospitalities, during the session, were altogether in the spirit and style peculiarly *Bostonian;* and if any of our readers are unacquainted with the significancy and force of this remark, we advise them not to rest satisfied with giving a loose rein to the imagination, but, if another like occasion offers, to inform themselves *in propria personæ*. All who may be practically acquainted with the meaning of the term, will be sure to avail themselves of any such occasion to refresh and perpetuate their knowledge.

"Well," the reader may now say, "what has the National Medical Association accomplished?" "I doubt not they who were so lucky as to be present, had a glorious time, but what, after all, did the meeting amount to; what real, permanent results will be brought about?"

The volume of Transactions will, in part, furnish an answer to inquiries like these—inquiries that have been repeatedly addressed to us in our intercourse with professional brethren. The published reports will (if we do not greatly err) be found to comprise a body of well digested information on the various branches of medical and surgical science, which will be read with pleasure and profit, and, as an exponent of medical inquiry and zeal in this country, will do us honor at home and abroad. We will not enlarge on this topic, as the volume (which we hope will be extensively circulated) will soon speak for itself.

It will be found that the Association will accomplish something for the cause of medical education. Doubtless the wishes of a few who desire peculiar and radical changes, under the illusory name of *reforms,* have been disappointed; and this furnishes, in our estimation, one of the best pledges of the usefulness of the Association. However attractive to the fancy, may be the destruction of existing systems and institutions, with the expectation of placing more perfect constructions on their ruins, wisdom and prudence would dictate a cautious policy under any circumstances. But for an Association endowed with no power to carry out its projects beyond its moral influence, a different policy would be the extreme of folly, inasmuch as injury or ruin would be inflicted upon itself alone. All must admit that evils are to be corrected, and improvements to be made, but how these desirable ends are to be best secured, is not so plain, and here there may exist wide differences of opinion. The Association, although invested with no executive authority to carry out its plans, furnishes an opportunity for the comparison of different views, and free discussion. It serves, also, as a means of concentrating, and giving utterance to the general voice of the profession. Compliance with its deliberate behests, albeit not compulsory, will yet be felt as a duty by the majority of members of the profession, in so far as compliance is practicable. It will be an useful engine of public medical opinion, while it is the faithful exponent of the general sentiment of the profession; and so long as it maintains this character, we feel assured that its course will be marked by temperateness and prudence.

One good result of these annual conventions is its influence on the public mind, exclusive of the medical Profession. The very fact of so large an assemblage of medical men for purposes of consultation and legislative deliberation on matters of science and education, tends to elevate our profession in the sense of the public, and especially in communities witnessing this congregation. This is an object of no mean importance.

Another good effect is, that by thus coming together, we acquire increased respect for each other, and greater regard for our common profession. Furnishing as it does, an occasion for forming agreeable and useful acquaintances, for mental and physical relaxation, and social enjoyment, each member returns to his accustomed duties with energies refreshed, and zeal renewed.

We might easily amplify on these, and other advantages, but this article is already unduly extended. In conclusion, we will remark, that we have occasionally heard expressions, depreciatory of the benefits of the Association, but, in every instance, such expressions have been uttered by those who have not been present at any of its meetings.

So far as we are conversant with the opinions of those, who have participated in the pleasures and usefulness of all, or any of these occasions, they are, without any exception, warmly in its favor. If this be the case generally with respect to those who have been present, it only remains for those who have not yet attended, to make the experiment, and the Association will necessarily become firmly fixed in the respect and attachment of the entire Profession.

